# Phytochemistry, Ethnopharmacological Uses, Biological Activities, and Therapeutic Applications of *Cassia obtusifolia* L.: A Comprehensive Review

**DOI:** 10.3390/molecules26206252

**Published:** 2021-10-15

**Authors:** Md Yousof Ali, Seongkyu Park, Munseog Chang

**Affiliations:** 1 Department of Physiology and Pharmacology, Hotchkiss Brain Institute and Alberta Children’s Hospital Research Institute, Cumming School of Medicine, University of Calgary, Calgary, AB T2N 4N1, Canada; mdyousof.ali@ucalgary.ca; 2 Department of Prescriptionology, College of Korean Medicine, Kyung Hee University, 26, Kyunghee dae-ro, Dongdaemun-gu, Seoul 02447, Korea; comskp@khu.ac.kr; 3 Qgenetics, Seoul Bio Corporation Center, 504, 23 Kyunghee Dae-ro, Dongdaemun-gu, Seoul 02447, Korea

**Keywords:** anthraquinones, antidiabetic, antimicrobial, *Cassia obtusifolia* L., hepatoprotection, neuro protection

## Abstract

*Cassia obtusifolia* L., of the Leguminosae family, is used as a diuretic, laxative, tonic, purgative, and natural remedy for treating headache, dizziness, constipation, tophobia, and lacrimation and for improving eyesight. It is commonly used in tea in Korea. Various anthraquinone derivatives make up its main chemical constituents: emodin, chrysophanol, physcion, obtusifolin, obtusin, au rantio-obtusin, chryso-obtusin, alaternin, questin, aloe-emodin, gluco-aurantio-obtusin, gluco-obtusifolin, naphthopyrone glycosides, toralactone-9-β-gentiobioside, toralactone gentiobioside, and cassiaside. *C*. *obtusifolia* *L*. possesses a wide range of pharmacological properties (e.g., antidiabetic, antimicrobial, anti-inflammatory, hepatoprotective, and neuroprotective properties) and may be used to treat Alzheimer’s disease, Parkinson’s disease, and cancer. In addition, *C*. *obtusifolia* L. contributes to histamine release and antiplatelet aggregation. This review summarizes the botanical, phytochemical, and pharmacological features of *C*. *obtusifolia* and its therapeutic uses.

## 1. Introduction

*Cassia* (family Caesalpiniaceae) is a large tropical genus with ~600 species of herbs, shrubs, and trees. *Cassia obtusifolia* (sicklepod) Linn., a member of the genus Cassia (Leguminosae), is a well-known traditional Chinese medicinal plant. It belongs to the medically and economically important family Leguminosae (syn. Fabaceae; subfamily Caesalpinioideae). *C*. *obtusifolia* L. is found mainly in China, Korea, India, and the western tropical regions. It is an annual semi-shrubby herb that ranges in height from ~0.5 to 2 m. It has two or three pairs of round-tipped leaflets with one to three flowers on a short axillary peduncle with pedicels up to 2 cm; the yellow petals (0.8–1.5 cm) wilt by midday. The pods are linear (up to 20 cm in length), curve gently downward, and contain numerous shiny, dark brown seeds (~0.5 cm in length). The seeds of *C*. *obtusifolia* L. are rhomboidal or slightly flat, with linear concave ramps on each side. *Cassia tora* L. is considered synonymous with *C*. *obtusifolia* L., but differs in its botanical and morphological characteristics [1,2]. The main distinguishing morphological feature between the two is the seed coat, which is marked with an obliquely symmetrical dented line on each side of the rib (*C*. *obtusifolia* L.) or has broad bands on both sides of the rib (*C*. *tora* L.).

*Cassia* species are of medicinal interest because of their therapeutic value in traditional medicine. The dry seeds are processed as a crude drug for clinical use or as a dietary supplement. The cultured plants are important sources of Semen Cassiae-derived commercial products in the market. *C*. *obtusifolia* L. seeds are a well-known medicinal plant in East Asia and are consumed as food to clear liver heat, sharpen vision, lubricate the intestines, and promote bowel movement [3]. In Korea, dried and roasted Cassia seeds are frequently used in brewing tea. In traditional oriental and Chinese (Juemingzi in Chinese) medicine, *C*. *obtusifolia* L. has been used to treat lacrimation, headaches, dizziness, and constipation [3,4]. *C*. *obtusifolia* L. has several pharmacological properties, including antiplatelet aggregation, antidiabetic, antimicrobial, anti-inflammatory, hepatoprotective, and neuroprotective activities, and may be used to treat Alzheimer’s disease, Parkinson’s disease, and cancer [5,6,7,8,9,10,11,12]. It also contributes to histamine release and antiplatelet aggregation. The whole plant, as well as its roots, flowers, leaves, seeds, and pods, possesses medicinal properties. A summary of the ethnomedicinal uses of different parts of the plant is provided in Table 1. This review herein summarizes progress regarding the chemical analysis of *C*. *obtusifolia* L., primarily focusing on the development of the phytochemistry, botanical aspects, ethnopharmacological, and pharmacological effects of *C*. *obtusifolia* L. *C*. *obtusifolia* L. species are rich sources of different types of anthraquinones and naphthopyrone derivatives that exhibit a number of biological activities and may potentially impact human health. Unfortunately, *C*. *obtusifolia* L. has not been developed as a pharmaceutical agent. The main objective of this review is to present a summary of the studies published to date on this promising plant, with a solid platform to design and conduct clinical studies. This paper reviews the phytochemical and pharmacological activities of *C*. *obtusifolia* L. and discusses its potential uses as a human food source and/or a pharmacological agent.

## 2. Phytochemistry

**Figure 1:**
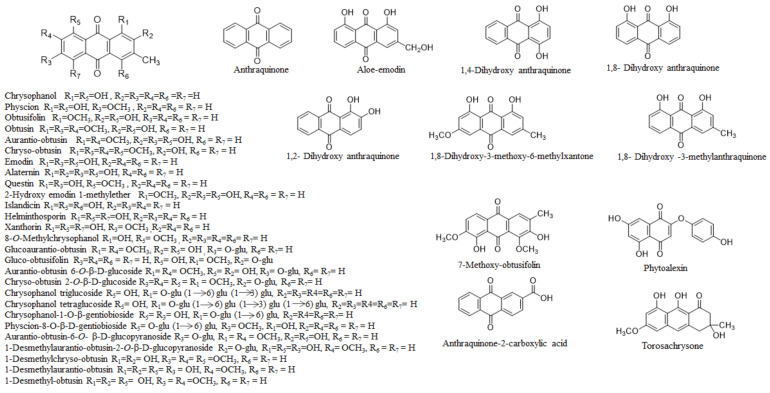

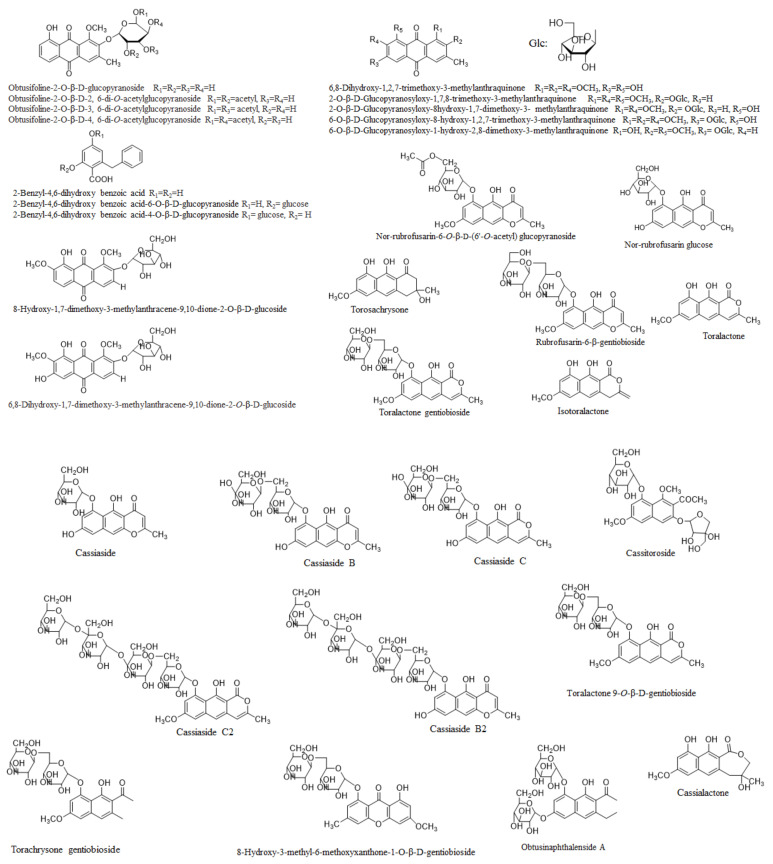
Chemical structures of the main compounds present in *Cassia obtusifolia* L.

Several classes of bioactive metabolites have been identified from *C*. *obtusifolia* L., including anthraquinones, terpenoids, flavonoids, and lipids [1,10,19]. The main plant chemicals include anthraquinone, emodin, chrysophanol, physcion, obtusifolin, obtusin, aurantio-obtusin, chryso-obtusin, alaternin, questin, aloe-emodin, gluco-aurantio-obtusin, gluco-obtusifolin, chrysophanol-2-*O*-tetraglucoside, chrysophanol-2-*O*-triglucosides, and chryso-obtusin-2-glucoside [2,5,6,7,8,9,10,11,12,19]. Other components include naphthopyrone glycosides, toralactone-9-β-gentiobioside, toralactone gentiobioside, cassiaside, rubrofusarin-6-*O*-gentiobiosideol, rubrofusarin-6-β-gentiobioside, cassiaside C, cassiaside B2, cassiaside C2, xanthones (1,8-dihydroxy-3-methoxy-6-methylxanthone, isogentisin, 1,7-dihydroxy-3-methylxanthone, euxanthone, 1,3,6-trihydroxy-8-methylxanthone), triterpenoids (lupeol, betulinic acid, α-amyrin, sterols, polyketide, steroids, fatty esters), and toralactone [1,17]. The chemical structures of the main compounds are presented in Figure 1. Research on *C*. *obtusifolia* L. reveals that the nature and number of phytochemicals vary according to climate. Researchers have found that the whole *C*. *obtusifolia* L. plant (seeds, twigs, leaves, and roots) is rich in free and bound anthraquinones, although the quantities differ markedly. In general, anthraquinone content is higher in seeds and less abundant in other components. The following section discusses the phytochemical contents of the various plant parts.

### 2.1. The Whole Plant

Analysis of the whole *C*. *obtusifolia* L. plant indicates the presence of various anthraquinones and naphthopyrones: aloe-emodin, emodin, 1,2-dihydroxyanthraquinone, obtusin, chryso-obtusin, aurantio-obtusin, gluco-obtusifolin, gluco-aurantio-obtusin, gluco-chryso-obtusin, 1-desmethylaurantio-obtusin-2-*O*-β-d-glucopyranoside, 1-desmethyl-obtusin, aurantio-obtusin-6-*O*-β-d-glucopyranoside, 1-desmethylaurantio-obtusin, alaternin-1-*O*-β-d-glucopyranoside, chryso-obtusin-2-*O*-β-d-glucopyranoside, physicon-8-*O*-β-d-glucoside, obtusifolin, *O*-methyl-chrysophanol, emodin-1-*O*-β-gentio-bioside, chrysophanol-1-*O*-β-gentiobioside, chrysophanol-1-*O*-β-d-glucopyranosyl-(13)-β-d-glucopyranosyl-(1→6)-β-d-glucopyranoside, physcion-8-*O*-β-glucoside, 1,3-dihydroxy-8-methylanthraquinone, torosachrysone, 1-methylaurantio-obtusin-2-*O*-β-d-glucopyranoside, 1-desmethylchryso-obtusin, chrysophanic, acid, physcion, chrysophanol-10,10′-bianthrone, physcion-8-*O*-β-gentiobioside, and questin [20].

### 2.2. Seeds

*Cassia obtusifolia* seeds are composed of 1–2% anthraquinones, 5–7% fats, 14–19% protein, and 66–99% carbohydrates [21]. In addition to proteins and fats, the seeds also contain a gum of commercial interest [22]. As much as 41% of the seed is extractable [23]. Several anthraquinone compounds and glycosides have been isolated from the methanol extract of the seeds; examples include anthraquinone, chrysophanol, physcion, emodin, obtusifolin, obtusin, questin, chryso-obtusin, gluco-obtusifolin, aloe-emodin, alaternin, aurantio-obtusin, gluco-aurantio obtusin, chrysophanol tetraglucoside, 2-hydroxyemodin-1 methylether, chryso-obtusin-2-glucoside, chrysophanol triglucoside, 1,2-dihydroxyanthraquinone, 1,4-dihydroxyanthraquinone, 1,8-dihydroxyanthraquinone, 1,8-dihydroxy-3-methylanthraquinone, naphthopyrone glycoside, toralactone gentiobioside, cassiaside, and the naphthalene glycoside cassitoroside [7,10]. Torosachrysone and naphthalenic lactones, isotoralactone, cassialactone, three benzyl-β-resorcylates (2-benzyl-4,6-dihydroxy benzoic acid, 2-benzyl-4,6-dihydroxy benzoic acid-6-O-β-d-glucopyranoside, and 2-benzyl-4,6-dihydroxy benzoic acid-4-O-β-d-glucopyranoside), a new sodium salt of anthraquinone (sodium emodin-1-*O*-*β*-gentiobioside), chrysophanol-1-*O*-*β*-d-glucopyranosyl-(1–3)-*β*-d-glucopyranosyl-(1–6)-*β*-d-glucopyranoside, rubrofusarin-6-*O*-*β*-d-gentiobioside, obtusifolin-2-*O*-*β*-d-glucopyranoside, aurantio-obtusin-6-*O*-*β*-d-glucopyranoside, physcion-8-*O*-*β*-d-glucopyranoside,1-hydroxyl-2-acetyl-3,8-dimethoxy-6-*O*-*β*-d-apiofuranosyl-(1–2)-*β*-d-glucosylnaphthalene, toralactone-9-*O*-*β*-d-gentiobioside, and rubrofusarin-6-*O*-*β*-d-apiofuranosyl-(1–6)-*O*-*β*-d-glucopyranoside have also been isolated from *C*. *obtusifolia* L. seeds [24,25,26]. In addition, three acetylated anthraquinone glycosides (obtusifoline-2-*O*-β-d-2,6-di-*O*-acetylglucopyranoside, obtusifoline-2-*O*-β-d-3,6-di-*O*-acetylglucopyranoside, and obtusifoline-2-*O*-*β*-d-4,6-di-*O*-acetylglucopyranoside) have been isolated from the ethanolic extract of the seeds [27]. Recently, Pang et al. [28,29] have isolated four new compounds from the seeds of *C. obtusifolia* obtusifolin-2-*O*-*β*-d-(6′-*O*-*α*, *β*-unsaturated butyryl)-glucopyranoside, *epi*-9-dehydroxyeurotinone-*β*-d-glucopyranoside, obtusinaphthalenside A, and obtusinaphthalenside B. Feng et al. [30] also purified various monosaccharides, and polysaccharides from the water extract of *C. obtusifolia* L.

### 2.3. Leaves

The leaves of *C. obtusifolia* L. contain anthraquinones, xanthones, polyketide, steroids, triterpenoids, and fatty esters [17]. The methanol extract of the leaves contains aloe emodin, emodin, 1,8-dihydroxy-3-methoxy-6-methylxantone, euxanthone, chrysophanol, physcion, 1,2,8-trihydroxy-6,7-dimethoxyanthraquinone,1,7-dihydroxy-3-methoxyxanthone,1,5-dihydroxy-3-methoxy-7-methylanthraquinone,3,7-dihydroxy-1-methoxyxanthone,1-*O*-methylchrysophanol, 8-*O*-methylchrysophanol, 1,3,6-trihydroxy-8-methylxanthone, 1-hydroxy-7-methoxy-3-methylanthraquinone, and obtusifolin. The ethyl acetate extract contains (4R*,5S*,6*E*,8*Z*)-ethyl-4-([*E*]-but-1-enyl)-5-hydroxypentdeca-6,8-dienoate, (24*S*)-24-ethylcholesta-5,22(*E*),25-trien-3β-ol, (–)-acetoxy-9,10-dimethyl-1,5-octacosanolide, friedelin, stigmasterol, lupeol, and (*E*)-eicos-14-enoic acid [17]. A single phytoalexin was isolated and purified from 12- to 14-day-old leaves [31].

### 2.4. Roots

The hairy roots of *C*. *obtusifolia* L. contain betulinic acid, sitosterol, stigmasterol, anthraquinones, chrysophanol, physicon, 1-hydroxy-7-methoxy-3-methylanthraquinone, 8-*O*-methylchrysophanol, 1-*O*-methylchrysophanol, 1,2,8-trihydroxy-6,7-dimethoxyanthraquinone, emodin, iso-landicin, helminthosporin, obtusifolin, aloe-emodin, and xanthorin [20,32].

## 3. Bioactivity

**Figure 2:**
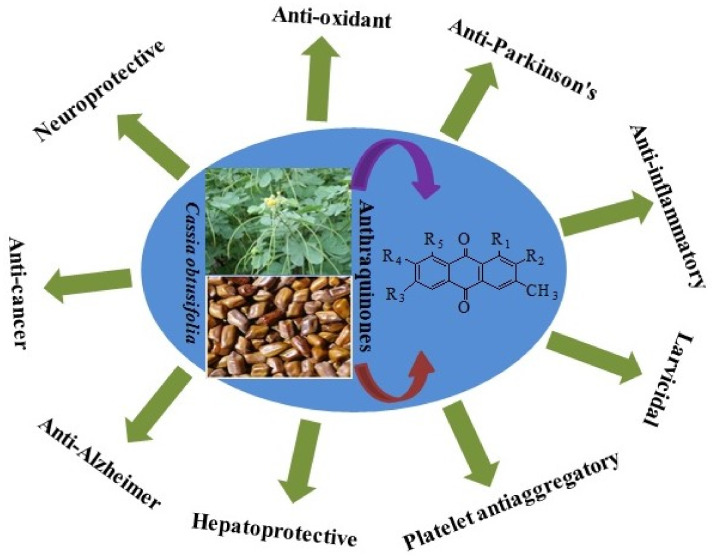
Different biological activities displayed by *Cassia obtusifolia*.

Numerous researchers have investigated the pharmacological activities of various *C*. *obtusifolia* L. extracts. Table 2 summarizes the pharmacological features that have been observed. They include: antidiabetic, anti-inflammatory, antimicrobial, antioxidant, hepatoprotective, neuroprotective, immune-modulatory, anti-Parkinson’s disease, anti-Alzheimer’s disease, and larvicidal properties. The anthraquinones and naphthopyrones isolated from *C*. *obtusifolia* L. are structurally diverse and exhibit multiple pharmacological properties, which suggests that these compounds contribute to its therapeutic effects (Table 3). *C*. *obtusifolia* L. and its major constituents display a vast number of biological activities (Figure 2). Natural products are highly promising sources for antioxidant and anti-inflammatory agents. A wide range of bioactive constituents of plants have antioxidant and anti-inflammatory activities. Based on various assay methods and activity indices, antioxidant or anti-inflammatory activities and nutraceutical and therapeutic effects of traditional Chinese medicines as well as the mechanisms underlying such activities and effects have been investigated. The generation of free radicals can result in damage to the cellular machinery. The seeds of *C. obtusifolia* L. are widely used in Chinese folk medicine and have been demonstrated to exhibit significant antioxidant and anti-inflammatory. Over the past century, natural products, especially anthraquinone compounds, have become valuable products for achieving chemical diversity in the molecules used for inflammation relief. In addition, COE has traditionally been used in Korea to treat eye inflammation, photophobia, and lacrimation.

### 3.1. Neuroprotective Activity

Various studies have demonstrated the direct neuroprotective activities of the *C*. *obtusifolia* L. seed extract (COE) and its major constituents (anthraquinones). More detailed studies are required to clarify the compositional features and neuroprotective activities of the anthraquinones. The ethanolic COE (25, 50, or 100 mg/kg) ameliorates scopolamine or bilateral common carotid artery occlusion (2VO)-induced memory impairment by inhibiting acetylcholinesterase [8]. COE (10 or 50 mg/kg/day) reduced memory impairment and neuronal damage caused by 2VO in a mouse model of transient global ischemia; it was suggested that the neuroprotective effects of COE are attributable to its anti-inflammatory properties resulting in decreased expression of inducible nitric oxide synthase (iNOX) and cyclooxygenase-2 (COX-2) and increased expression of the neurotrophic factors pCREB and BDNF [33]. Alaternin, the active compound in *C*. *obtusifolia* L., exhibits neuroprotective activity after transient cerebral hypoperfusion induced by bilateral common carotid artery occlusion. Administration of alaternin (10 mg/kg) prevented or reduced nitrotyrosine and lipid peroxidation, bilateral common carotid artery occlusion (BCCAO)-induced iNOS expression, and microglial activation [48]. Drever et al. [11] reported that ethanolic COE is neuroprotective against NMDA-induced calcium dysregulation and 3-nitropropionic acid-induced cell death in mouse hippocampal cultures. Recently, Paudel et al. [56] also reported that four major compounds (cassiaside, rubrofusarin gentiobioside, aurantio-obtusin, and 2-hydroxyemodin 1-methylether) exhibited neuroprotective effects; among them, aurantio-obtusin showed promising neuroprotective effects via targeting various G-protein-coupled receptors and transient brain ischemia/reperfusion injury C57BL/6 mice model.

#### 3.1.1. Anti-Alzheimer’s Disease Activity

The effects of the ethanolic extract of COE in Aβ-induced anti-Alzheimer’s disease (anti-AD) models have been reported. The mechanism of COE ameliorated Aβ-induced LTP impairment in acute hippocampal slices and prevented Aβ-induced GSK-3β activation [35]. Moreover, COE prevented microglial activation as well as iNOS and COX activation induced by Aβ in the hippocampus, and in vivo studies have indicated that COE ameliorated Aβ-induced object recognition memory impairment [35]. Two anthraquinones from *C*. *obtusifolia* L., obtusifolin and gluco-obtusifolin, improved scopolamine-induced learning and memory impairment in mice based on the passive avoidance and Morris water maze tests [49]. Obtusifolin (0.25, 0.5, and 2 mg/kg) and gluco-obtusifolin (1, 2, and 4 mg/kg) significantly reversed scopolamine-induced cognitive impairment on the passive avoidance test; obtusifolin (0.5 mg/kg) and gluco-obtusifolin (2 mg/kg) improved escape latencies, swimming times in the target quadrant, and crossing numbers in the zone where the platform previously existed on the Morris water maze test [49]. The anti-AD properties of COE may be attributed to its constituents, such as anthraquinones and naphthopyrone glycosides. The methanolic seed extract and its solvent-soluble fractions from *C*. *obtusifolia* L. were tested for their acetylcholinesterase (AChE) and butyrylcholinesterase (BChE) inhibitory activities using Elman’s method. Ethyl acetate and butanol fractions significantly inhibited AChE activity at a final concentration of 100 µg/mL, with IC_50_ values of 9.45 ± 0.44 and 9.87 ± 0.70 μg/mL, respectively. Butanol (IC_50_ = 7.58 ± 0.51 μg/mL) and ethyl acetate (IC_50_ = 16.09 ± 0.16 μg/mL) fractions exhibited potent inhibitory activity against BChE. Furthermore, butanol fraction (IC_50_ = 26.19 ± 0.72 μg/mL) significantly inhibited the β-secretase (BACE1) activity [10]. In addition, several anthraquinones (emodin, chrysophanol, physcion, obtusifolin, alaternin, questin, aloe-emodin) that displayed strong anti-AD activity by inhibiting AChE, BChE, and BACE1 enzymes were isolated from this plant [10]. Recently, Shrestha et al. [55] observed anti-AD effects of naphthopyrone and its glycosides including rubrofusarin, rubrofusarin 6-*O*-β-d-glucopyranoside, rubrofusarin 6-*O*-β-d-gentiobioside, nor-rubrofusarin 6-*O*-β-d-glucoside, isorubrofusarin 10-*O*-β-d-gentiobioside, and rubrofusarin 6-*O*-β-d-triglucoside by inhibiting AChE, BChE, and BACE1 enzymes. The use of AChE, BChE, and BACE1 inhibitors has been a promising treatment strategy for AD; therefore, *C*. *obtusifolia* may be an effective agent for treating AD.

#### 3.1.2. Prevention and Treatment of Parkinson’s Disease

A neuroprotective effect of COE was observed in both in vitro and in vivo models of Parkinson’s disease [34]. In PC12 cells, COE reduced cell damage induced by 100 µM 6-hydroxydopamine and inhibited the overproduction of reactive oxygen species, glutathione depletion, mitochondrial membrane depolarization, and caspase-3 activation at 0.1 to 10 µg/mL. In addition, COE displayed radical scavenging effects in DPPH and ABTS assays, which suggests that COE may be useful for treating Parkinson’s disease [34].

### 3.2. Hepatoprotective Activity

Few studies have demonstrated the hepatoprotective activities of COE [15]. Further studies are required to establish the hepatoprotective mechanisms of major COE anthraquinones. The protective effects of ethanolic COE against the cytotoxicity induced by CCl_4_ liver in mice were evaluated by assessing aminotransferase activities, histopathological changes, hepatic and mitochondrial antioxidant indices, and cytochrome P450 2E1(CYP2E1) activity. Administration of COE (0.5, 1, 2 g/kg) markedly reduced ALT and AST release, Ca^2+^-induced mitochondria membrane permeability transition, and CYP2E1 activity. In addition, COE significantly reduced hepatic and mitochondrial malondialdehyde levels, increased hepatic and mitochondrial glutathione levels, and restored superoxide dismutase, glutathione reductase, and glutathione S-transferase activities [15]. Meng et al. [38] reported the hepatoprotective effects of ethanolic COE on non-alcoholic fatty liver disease (NAFLD). Administration of COE (0.5, 1, 2 g/kg) markedly reduced the levels of AST, ALT, TG, TC, TNF-a, IL-6, IL-8, and MDA. COE treatments also increased the levels of SOD, GSH, and the expression of LDL-R mRNA [38]. Seo et al. [12] observed hepatoprotective effects of ethanolic COE and its components (e.g., toralactone glycoside) in *t*-BHP-induced cell death in HepG2 cells. *Cassia* anthraquinones, aurantio-obtusin, and obtusifolin also protected against tacrine-induced cytotoxicity in HepG2 cells [36]. Recently, Ali et al. [37] investigated the hepatoprotective effects of different soluble fractions of methanolic derived COE and its active components in *t*-BHP-induced oxidative stress in HepG2 cells. The possible mechanism was that alaternin, aloe emodin, and cassiaside potently scavenge ROS in *t*-BHP-induced HepG2 cells and the decrease in ROS generation parallels the up-regulation of glutathione (GSH). Very recently, Paudel et al. [57] investigated the hepatoprotective activity of an anthraquinone (1-desmethylaurantio-obtusin 2-*O*-*β*-d-glucopyranoside) and two naphthopyrone glycosides (rubrofusarin 6-*O*-β-d-apiofuranosyl-(1→6)-*O*-*β*-d-glucopyranoside and rubrofusarin 6-*O*-β-gentiobioside) isolated from the butanol fraction of COE in the *t*-BHP-induced oxidative stress in HepG2 cells through up-regulated HO-1 via the nuclear factor erythroid-2-related factor 2 (Nrf2) activation and modulation of the JNK/ERK/MAPK signaling pathway.

### 3.3. Anti-Inflammatory and Antioxidant Activity

COE has traditionally been used in Korea to treat eye inflammation, photophobia, and lacrimation. Pretreatment with the aqueous extract of *C*. *obtusifolia* L. inhibited interleukin (IL)-6 and cyclooxygenase-2 (COX-2) and reduced the activation of transcription nuclear factor-kB p65 in colon tissues treated with dextran sulfate sodium [40]. Two major anthraquinones from *C*. *obtusifolia*, obtusifolin and gluco-obtusifolin, reduced neuropathic and inflammatory pain [40]. Pro-inflammatory cytokines (e.g., TNF-*α*, IL-1*β*, IL-6) and activation of NF-kB have been strongly implicated in the initiation and development of inflammatory and neuropathic pain, and the administration of obtusifolin and gluco-obtusifolin (1 and 2 mg/kg) significantly inhibited this upregulation. This finding suggests that obtusifolin and gluco-obtusifolin inhibited the overexpression of spinal TNF-α, IL-1β, IL-6, and NF-κB p65 associated with inflammatory and neuropathic pain, which involves the regulation of neuroinflammatory processes and the neuroimmune system [51]. In another study, water-extracted polysaccharides (CP) from the whole seeds of *C*. *obtusifolia* L. and its two subfractions CP-30 and CP-40 were obtained. CP, CP-30, and CP-40 possessed immunomodulation activity by promoting phagocytosis and stimulating the production of nitric oxide (NO) and cytokines TNF-α and IL-6 [41]. Methanolic COE was investigated for antioxidant and health-relevant functionality. The extract exhibited 1292 mM Fe[II] per 1 mg/mL extract of antioxidant power, 49.92% inhibition of β-carotene degradation, 65.79% of scavenging activity against DPPH, and 50.78% of superoxide radicals (at a concentration 1 mg/mL). These antioxidant properties may be attributed to the total free phenolic content of the raw seeds, which was 13.33 ± 1.73 g catechin equivalent/100 g extract [14]. Recently, Kwon et al. [58] investigated the anti-inflammatory activity of major anthraquinone derivatives; among them, aurantio-obtusin inhibited iNOS expression without affecting iNOS enzyme activity and down-regulation mechanisms included interruption of the JNK/IKK/NF-κB activation and proinflammatory cytokine production from the lung-related cells. Additionally, aurantio-obtusin also dose-dependently (10 and 100 mg/kg) inhibited the inflammatory responses in a mouse model of airway inflammation, LPS-induced acute lung injury. Very recently, Hou et al. [54] reported anti-inflammatory activity by decreasing the production of NO, PGE2, and inhibiting iNOS, COX-2, TNF-α, and IL-6. Additionally, there was a reduction in the LPS-induced activation of nuclear factor-κB in RAW264.7 cells [54].

### 3.4. Antimicrobial Activity

Because many bacterial and fungal strains are resistant to a wide variety of antibiotics, medicinal plants have been studied for their potential antimicrobial properties. COE was active against several different microbes (*Bifidobacterium adolescentis*, *B*. *bifidum*, *B*. *longum*, *B*. *breve*, *Clostridium perfringens*, *Escherichia coli*, *Lactobacillus casei*). Isolated 1,2-dihydroxyanthraquinone strongly inhibited the growth of *C*. *perfringens* and *E*. *coli* and promoted the growth of *B*. *bifidum* [7]. The *C*. *obtusifolia* L. leaf extract in petroleum ether and chloroform showed sensitivity against *E*. *faecalis* (minimal inhibitory concentration [MIC] 0.2725 mg/mL), whereas ethanol extracts showed sensitivity against *A*. *fumigatus* (MIC 0.3116 mg/mL). Similarly, stem extracts of *C*. *obtusifolia* L. in petroleum ether showed sensitivity against *E*. *faecalis* (MIC 0.407 mg/mL), ethanol extracts showed sensitivity against *E*. *faecalis* (MIC 0.3009 mg/mL), and chloroform extracts showed sensitivity against *E*. *faecalis* MIC 0.4946 mg/mL [18]. The whole plant extract of *C*. *obtusifolia* significantly inhibited the growth of *Staphyloccocus aureus* MRSA8 (MIC 64 μg/mL), *E*. *coli* AG100 (MIC 256 μg/mL), *Pseudomonas aeruginosa* PA01 (MIC 256 μg/mL), *Enterobacter aerogenes* EA289 (MIC 289 μg/mL), and *Klebsiella pneumoniae* KP55 MIC 256 μg/mL [42]. Phytoalexin 2-(phydroxyphenoxy)-5,7-dihydroxychromone isolated from *C*. *obtusifolia* L. exhibited strong antifungal activity [31]. The *C*. *obtusifolia* L. root extract and its constituents exhibited strong antibacterial activity. Emodin, 2,5-dimethoxybenzoquione, questin, isotoralactone, and toralactone exhibited strong antibacterial activity against *S*. *aureus* 209P (MICs 4.5, 19, 25, and 3 µg/mL, respectively) and *E. coli NIHJ* MICs 25, 50, 50, 12, and 5.5 µg/mL, respectively [46].

### 3.5. Antidiabetic Activity

Two key enzymes, protein tyrosine phosphatase 1B (PTP1B) and α-glucosidase, are effective in treating diabetes mellitus. The effects of methanolic COE revealed inhibitory activities against PTP1B and α-glucosidase. Out of 15 anthraquinones from the extract, compounds with alaternin, physcion, chrysophanol, emodin, obtusin, questin, chryso-obtusin, aurantio-obtusin, 2-hydroxyemodin-1 methylether, gluco-obtusifolin, gluco-aurantio obtusin, and naphthalene glycoside aloe-emodin exhibited the highest inhibitory activities against PTP1B and α-glucosidase in vitro [9]. The effects of alaternin and emodin on the stimulation of glucose uptake by insulin-resistant human HepG2 cells were examined at concentrations ranging from 12.5 to 50 µM and 3.12 to 12.5 µM, respectively. In another study, five new anthraquinones were isolated from ethanol seed extracts of *C*. *obtusifolia* L. and evaluated for their antidiabetic activities through the inhibition of α-glucosidase in vitro [39]. Obtusifolin isolated from *C*. *obtusifolia* L. may have an antihyperlipidemic effect; an intraperitoneal obtusifolin injection reduced blood lipid levels in streptozotocin-induced diabetic rats [59]. Results from another study indicated that oral administration of obtusifolin significantly reversed the changes induced by hyperlipidemia in body weight, total cholesterol, triglycerides, low-density lipoprotein cholesterol, and high-density lipoprotein cholesterol; increased serum superoxide dismutase, and nitric oxide, and reduced malondialdehyde [50].

Recently, two new naphthalenic lactone glycosides(3*S*)-9,10-dihydroxy-7-methoxy-3-methyl-1-oxo-3,4-dihydro-1H-benzo[g]isochromene-3-carboxylic acid 9-*O*-β-d-glucopyranoside and (3*R*)-cassialactone 9-*O*-β-d-glucopyranoside were isolated from seeds of *C. obtusifolia* L. that showed significant inhibitory activities against the formation of advanced glycation end-products (AGEs) with IC_50_ values of 11.63 and 23.40 µM, respectively [60].

### 3.6. Antiplatelet Aggregation Inhibitory Activity

Ethanolic COE and three major anthraquinones (aurantio-obtusin, chryso-obtusin, and emodin) demonstrated inhibitory activity against ADP (adenosine 5′-diphosphate), arachidonic acid (AA), or collagen-induced platelet aggregation [47]. Methanolic COE and different solvent soluble fractions, including normal butanol (*n*-BuOH) and dicloromethane (CH_2_Cl_2_), exhibited antiplatelet aggregation activities. Furthermore, 17 anthraquinones, including gluco-obtusifolin, gluco-aurantio-obtusin, obtusifolin, and gluco-chryso-obtusin, were identified as active antiplatelet aggregation components [5].

### 3.7. Anticancer Activity

Polysaccharide COB1B1S2 and its sulfated derivative COB1B1S2-Sul were isolated from an alkaline COE. Human hepatocellular carcinoma cell lines Bel7402, SMMC7721, and Huh7, as well as HT-29 and Caco-2, were used to evaluate the anticancer effects of COB1B1S2 and COB1B1S2-Sul [61]. COB1B1S2 had a weak inhibitory effect on Bel7402, Huh7, HT-29, as well as Caco-2 cells. By contrast, COB1B1S2-Sul significantly inhibited the growth of all cell lines, particularly Bel7402 cells at 250 µg/mL; the inhibition ratio was 61.7% [62]. Three acetylated benzyl-beta-resorcylate glycosides (2-benzyl-4,6-dihydroxy benzoic acid-6-O-[2,6-*O*-diacetyl]-d-glucopyranoside, 2-benzyl-4,6-dihydroxy benzoic acid-6-*O*-[3,6-*O*-diacetyl]-d-glucopyranoside, and 2-benzyl-4,6-dihydroxy benzoic acid-6-*O*-[4,6-*O*-diacetyl]-d-glucopyranoside) were isolated from seeds of *C*. *obtusifolia* and exhibited significant cytotoxicity against a human hepatoblastoma cell line, with IC_50_ values of 4.6, 5.0, and 4.3 µg/mL, respectively [62]. In addition, 12 compounds were isolated from seeds of *obtusifolia* and their anticancer activities evaluated in multiple cancer cell lines [52]. 8-Hydroxy-1,7-dimethoxy-3-methylanthracene-9,10-dione-2-*O*-β-d-glucoside was active against HCT-116, A549, HepG2, SGC7901, and LO2 cell lines, with IC_50_ values of 4.5, 7.6, 22.8, 20.7, and 18.1 µg/mL, respectively. 6,8-Dihydroxy-1,7-dimethoxy-3-methylanthracene-9,10-dione-2-*O*-β-d-glucoside was only weakly active against HCT-116 (IC_50_, 43.0 µg/mL). 1-Desmethylobtusin had moderate cytotoxicity against HCT-116, A549, and SGC7901cell lines, with IC_50_ values of 5.1, 10, and 25.4 µg/mL, respectively. Chryso-obtusin showed significant cytotoxic activity against HCT-116, A549, SGC7901, and LO2 cell lines, with IC_50_ values of 10.5 to 15.8 µg/mL. Obtusin was moderately active against HCT-116, A549, and SGC7901 cell lines, with IC_50_ values of 13.1, 29.2, and 15.2 µg/mL, respectively. Aurantio-obtusin was moderately active against HCT-116, A549, SGC7901, and LO2 cell lines, with IC_50_ values of 18.9 to 22.0 µg/mL. Chryso-obtusin-2-O-β-d-glucopyranoside was selectively cytotoxic against HCT-116, A549, HepG2, SGC7901, and LO2 cell lines, with IC_50_ values of 5.8 to 14.6 µg/mL. Finally, aurantio-obtusin-6-O-β-d-glucopyranoside was weakly cytotoxic against HCT-116 and SGC7901, with IC_50_ values of 31.1 and 23.3 µg/mL, respectively [52].

### 3.8. Larvicidal Activity

The larvicidal activity of methanol COE against early fourth-stage larvae of *Aedes aegypti* and *Culex pipiens pallens* was investigated [43]. At 200 ppm, extracts of *C*. *obtusifolia* L. caused more than 90% mortality in larvae of *Ae*. *aegypti* and *Cx*. *pipiens pallens*. At 40 ppm, extracts of *C*. *obtusifolia* L. caused 51.4% and 68.5% mortality in fourth-stage larvae of *Ae*. *aegypti* and *Cx*. *pipiens pallens*, respectively. Larvicidal activity of *C*. *obtusifolia* extract at 20 ppm was significantly reduced [43]. In another study, COE obtained in different fractions showed mosquito larvicidal activity against fourth instar larvae of *A*. *aegypti*, *Aedes togoi*, and *Cx*. *pipiens pallens* [44]. However, the chloroform fraction of *C*. *obtusifolia* extracts exhibited a strong larvicidal activity of 100% mortality (at a concentration 25 mg/L), and the isolated active compound emodin showed strong larvicidal activity, with LC_50_ values of 1.4, 1.9, and 2.2 mg/L against *C*. *pipiens pallens*, *A*. *aegypti*, and *A*. *togoi*, respectively [44]. The ethanolic leaf extract of *C*. *obtusifolia* L. was also investigated for larvicidal and oviposition deterrence effects against late third instar larvae of *Anopheles stephensi* [45]. Extracts from the leaf displayed significant larvicidal activity, with LC_50_ and LC_90_ values of 52.2 and 108.7 mg/L, respectively (at concentrations of 25 mg/L). In addition, the oviposition study indicated that different concentrations of leaf extract greatly reduced the number of eggs deposited by gravid *A*. *stephensi*. At concentrations of 100, 200, 300, and 400 mg/L, the maximum percentages of effective repellency against oviposition were 75.5%, 83.0%, 87.2%, and 92.5%, respectively [45].

### 3.9. Other Activities

The methanol extract of *C*. *obtusifolia* L. and its isolated naphthopyrones cassiaside B2 and cassiaside C2 inhibited histamine release from rat peritoneal exudate mast cells induced by antigen–antibody reaction [6]. The anti-angiogenic activity of two polysaccharides, COB1B1S2 and COB1B1S2-Sul, from *C*. *obtusifolia* L. seeds was evaluated by tube formation of HMEC-1 cells on Matrigel. COB1B1S2 at 50 or 100 µg/mL did not impair tube formation, but COB1B1S2-Sul at 50 or 100 µg/mL significantly disrupted tube formation; even at 50 µg/mL, COB1B1S2-Sul could potentially completely inhibit tube formation in HMEC-1 cells [61]. Water-soluble polysaccharides (WSPs) from *C*. *obtusifolia* L. (pectic polysaccharides and hemicellulose) were identified. These WSPs reduced pancreatic α-amylase activity by 20.5% and 28.9% (at concentrations of 20 and 80 mg/mL, respectively), reduced pancreatic lipase activity by about 18.9% (at a concentration of 80 mg/mL), and increased protease activity 5- to 7-fold (at concentrations of 20 and 80 mg/mL, respectively). These WSPs were also able to bind bile acids and reduce the amount of cholesterol available for absorption [63]. The simultaneous determination and pharmacokinetic study of seven anthraquinones (chrysophanol, emodin, aloe-emodin, rhein, physcion, obtusifolin, and aurantio-obtusin) in rat plasma after oral administration of *C*. *obtusifolia* L. extract was investigated and may help to explain the bioactivity and clinical applications of *C*. *obtusifolia* L. [64]. The effects of COE and its anthraquinones on muscle mitochondrial function were evaluated in vivo in rats and in vitro using mitochondrial energy metabolism models. The organic extract of *C*. *obtusifolia* L. and emodin significantly inhibited NADH: cytochrome c oxidoreductase activity of bovine heart mitochondrial particles and NADH: coenzyme Q oxidoreductase activity of porcine heart mitochondrial NADH dehydrogenase and exhibited protective effects of coenzyme Q against enzyme inhibition by anthraquinones [65]. Inhibition of trypsin activity by *C*. *obtusifolia* L. seeds was investigated [66]. A Kunitz-type trypsin inhibitor showed strong resistance against the midgut trypsin-like protease of *Pieris rapae*. In addition, a trypsin inhibitor gene (*CoTI1*) was isolated from *C*. *obtusifolia* L. and exhibited dominant inhibitory activities against trypsin and trypsin-like proteases from *Helicoverpa armigera*, *Spodoptera exigua*, and *Spodoptera litura* [67]. Moreover, Dong et al. [68], has been also reported that Cassia semen (*C. obtusifolia* and *C. tora*) and its major constituents possesses a wide spectrum of pharmacological properties.

## 4. Conclusions and Perspectives

As presented in this review, pharmacological studies on *C*. *obtusifolia* L. and its putative active compounds, especially anthraquinones and naphthopyrone, support that several biological activities of *C*. *obtusifolia* can potentially impact human health. Anthraquinones and naphthopyrone can be effectively isolated and purified from *C*. *obtusifolia* seeds, leaves, root and its whole plant with various extraction analytical methods, mainly separation-based methods using TLC, HPLC, high-speed counter-current chromatography (HSCCC), and column chromatography (silica gel, reverse-phase, and Sephadex). The semi-shrubby herb *C*. *obtusifolia* L., which belongs to the family Leguminosae, has gained popularity because of its medicinal and historical importance. It has been widely used in traditional medicine to treat headaches, dizziness, dysentery, and eye disease. In addition, *C*. *obtusifolia* L. is important to the food industry and possesses a wide spectrum of pharmacological properties (e.g., anti-allergic, antidiabetic, anti-inflammatory, antimicrobial, antioxidant, hepatoprotective, neuroprotective, anti-Alzheimer’s disease, antiplatelet aggregation, and larvicidal activities) that are associated with its diverse chemical constituents (e.g., anthraquinones, naphthopyrone, terpenoid, flavonoid, polysaccharides, and lipids). The number of modern studies on bioactive compounds is increasing in biomedicine, suggesting that these compounds might have great medical significance in the future. Although the bioactivities of seed extracts or compounds isolated from *C*. *obtusifolia* L. have been substantiated using in vitro and in vivo studies, the mechanisms of action remain unknown. Thus, there are still opportunities and challenges for research of seed extracts or compounds. Therefore, additional studies are required before *C*. *obtusifolia* L. and its components can be considered for further clinical use. In conclusion, *C*. *obtusifolia* L. is an edible medicinal plant that is important to the food industry and has a wide range of potential pharmacological uses. This review presents a summary of studies published to date on this promising plant.

## Figures and Tables

**Table 1:** Ethnomedicinal importance of *Cassia obtusifolia*.

Sr. No.	Plant Part Used	Ethnomedicinal Use
1	Whole plant	In traditional Oriental medicine, the whole plant of *C. obtusifolia* has been used for treatment of Laxative, eye infections, diarrhea, urinary tract infections, gingivitis, fever, and cough remedy [13].
2	Roots	Root is considered bitter, tonic, stomachic and is antidote against snake bite. Other uses are in treatment of fungal diseases, worm infection, abdominal tumors, bronchitis, and asthma. The roots of *C. obtusifolia* are also usually crushed, mixed with lime juice, and applied to ringworms [14].
3	Seeds	The seeds of *C. obtusifolia* are used to treat dizziness and to benefit the eyes by anchoring and nourishing the liver. The dried and roasted seeds are also used as brew a tea. Seeds of *C. obtusifolia* were also used for the treatment of headache, ophthalmic diseases, constipation, hypertension, and hyperlipidemia. In Korea, the hot extract of seeds is taken orally for protection of liver [10,15].
4	Leaves	*C. obtusifolia* leaves and pods have been widely used as purgatives and laxatives. In Indian traditional ayurveda system, the leaves and Pods are used as digestible, laxative, diuretic, stomachic, antipyretic, improves the appetite, biliousness, blood diseases, burning sensation, leprosy, bronchitis, piles, and leucorrhoea [16,17].
5	Stem bark	In Indian traditional ayurveda system, Stem bark extract is used for various skin ailments, rheumatic diseases, and as laxative [18].
6	Pods and fruits	Pods are used in dysentery, in eye diseases and pains in the joints. The unripe fruits are also cooked and eaten [14].

**Table 2:** Pharmacological activities of *Cassia obtusifolia* extracts.

Pharmacological Activity	Part of Plant	Type of Extract	In Vivo/ In Vitro	Model	Administration (In Vivo)	Dose Range	Active Concentration	Reference
**Neuroprotective Activity**	Seeds	85% EtOH ext.	In vivo	Ameliorate scopolamine or 2VO-induced memory impairments by inhibiting AChE	Oral	25–100 mg/kg	50 mg/kg	[8]
	Seeds	85% EtOH ext.	In vivo	Neuroprotection by inhibition of pro-inflammatory genes iNOX, and COX-2, and increased neurotrophic factor expression of pCREB and BDNF	Oral	10, 50 mg/kg	50 mg/kg	[33]
	Seeds	85% EtOH ext.	In vitro	Reduced Aβ toxicity and maintenance of Ca^2+^ dysregulation and excitotoxicity, mitochondrial dysfunction in primary hippocampal cultures	-	0.1–10 µg/mL	1, 10 µg/mL	[11]
	Seeds	EtOH ex.	In vivo	protected the dopaminergic cells against 6-OHDA- and MPP+-induced neurotoxicities in primary mesencephalic cultures and in a mouse model in PD	Intraperitoneal injection	0.1–10 µg/mL for DA, 50 mg/kg mouse	0.1, 1 µg/mL 50 mg/kg	[34]
	Seeds	EtOH ext.	In vitro	Inhibited cell loss against 6-OHDA-induced DA neural toxicity by an anti-oxidant and anti-mitochondrial-mediated apoptosis mechanism in PC12 cells.	-	0.1–10 µg/mL 1000 µg/mL for DPPH, ABTS	1 µg/mL ROS, 10 µg/mL GSH, 75% Casp-3, 92%-DPPH, 85% ABTS	[35]
	Seeds	MeOH ext. EtOAc fr. CH_2_Cl_2_ fr. BuOH fr.	In vitro	Inhibitory activity against MAO-A, and MAO-B	-	0.25–120 µg/mL	EtOAc fr. exhibited greatest inhibitory IC_50_ = 20, and 56 µg/mL activity against MAO-A, and MAO-B	[36]
	Seeds	MeOH ext. EtOAc fr. CH_2_Cl_2_ fr. BuOH fr. H_2_O fr.	In vitro	Inhibitory activity against AChE, BChE, BACE1	-	0.4–120 µg/mL	IC_50_ = 9.45~29 µg/mL for AChE, IC_50_ = 7.58~49 µg/mL for BChE, IC_50_ = 26~96 µg/mL for BACE1	[10]
	Seeds	85% EtOH ext.	In vivo	Ameliorate Aβ-induced LTP impairment in the acute hippocampal slices and regulates GSK-3β, Akt signaling pathways through the inhibition of iNOS, COX expression	-	1 and 10 µg/mL	10 µg/mL	[35]
**Hepatoprotective Activity**	Seeds	MeOH ext.	In vitro	Protection against tacrine-induced hepatotoxicity in HepG2 cells	-	300 µg/mL	300 µg/mL	[36]
	Seeds	70% EtOH ext. EtOAc, CH_2_Cl_2_, BuOH, H_2_O fr.	In vitro	Protective effect against t-BHP-induced hepatotoxicity in HepG2 cells	-	10–100 µg/mL	EtOAc fr. showed most potent hepatoprotective activity (30 µg/mL)	[12]
	Seeds	EtOH ext.	In vivo	Hepatoprotective effects against CCl_4_-induced liver injury in mice	Intraperitoneal injection	0.5, 1, 2 g/kg	Reduced ALT and AST, Ca^2+^, MDA, and increased GSH, SOD, GR, GPx, GST, CYP2E1 (2 g/kg)	[15]
	seeds	EtOAc fr. CH_2_Cl_2_ fr. BuOH fr. H_2_O fr.	In vitro	Protective effect against *t*-BHP-induced hepatotoxicity in HepG2 cells	-	12.5–50 µg/mL	EtOAc fr. showed most potent hepatoprotective activity (50 µg/mL)	[37]
	Seeds	70% EtOH ext.	In vivo	(a) Significantly decreased the levels of AST, ALT, TG, TC, TNF-a, IL-6, IL-8 and MDA; (b) Increased the levels of SOD and GSH; (c) Significantly increased the mRNA expression levels of LDL-R	Oral	0.5–2 g/kg	(a) Dose-dependently decreased biomarkers at 0.5–2 g/kg; (b) Dose-dependently decreased at 0.5–2 g/kg; (c) Significantly increased the levels of LDL-R at 2 g/kg	[38]
**Anti-diabetic Activity**	Seeds	MeOH ext. EtOAc fr. CH_2_Cl_2_ fr. BuOH fr. H_2_O fr.	In vitro	Inhibitory activity against PTP1B and α-glucosidase	-	0.4–400 µg/mL for PTP1B, 0.16–400 µg/mL for α-glucosidase	MeOH ext. (IC_50_ = 14 µg/mL) and EtOAc fr. (IC_50_ = 74 µg/mL) exhibited greatest inhibitory activity against PTP1B and α-glucosidase	[9]
	Seeds	EtOH ext.	In vitro	Inhibitory activity against α-glucosidase	-	1000 µg/mL	20% inhibition of α-glucosidase (1000 µg/mL)	[39]
**Anti-inflammatory, Antioxidant, and Immune-modulatory Activities**	Roasted seeds	Hot H_2_O ext.	In vivo	Protection against dextran sulfate sodium (DSS)-induced colitis through the inhibition of (IL)-6, COX-2, NF-κB	Oral	1 g/kg	Significantly reduced clinical signs and the levels of inflammatory mediators (at concentration 1 g/kg)	[40]
	Seeds	H_2_O soluble polysaccharide fr.	In vitro	Increased immune-modulatory activity by promoting phagocytosis and stimulating the production of NO and cytokines TNF- and IL-6 on macrophage cell line RAW264.7	-	62.5–500 µg/mL	Stimulates NO, TNF- and IL-6 expression (250 µg/mL) and promotes phagocytic activity (500 µg/mL)	[41]
	Seeds	MeOH ext.	In vitro	DPPH, Fe [II], superoxide radicals scavenging activity and inhibit ß-carotene degradation	-	1 mg/mL	Inhibition 65.79% DPPH, 50.78% superoxide radical, 49.92% inhibit ß-carotene degradation,1292 mM Fe [II] inhibited (at 1 mg/mL)	[14]
**Antimicrobial Activity**	Seeds	MeOH ext. Hexane fr. EtOAc fr. CH_2_Cl_2_ fr. BuOH fr.H_2_O fr.	In vitro	Bifidobacterium adolescentis, B. bifidum, B. longum, B. breve, Clostridium perfringens, Escherichia coli, Lactobacillus casei	-	5 mg discs^−1^	CH_2_Cl_2_ fr, MeOH ext. and Hexane fr. exhibited the greatest antibacterial activity	[7]
	Leaf	Pet ether ext. EtOH ext. Chloroform ext.	In vitro	*Aspergilus fumigatus*, *Staphylococcus aureus*, *Enterococcus faecalis*, *E. coli*, *Klebsiella* sp., *Candia albicans*	-	0.6–1 mg/mL	Pet ether, chloroform ext. active against *C. albicans* (MIC 0.3524, and 0.4239 mg/mL), ethanol *E. faecalis* (MIC 0.2738 mg/mL)	[18]
	stem	Pet ether ext. EtOH ext. Chloroform ext.	In vitro	*Aspergilus fumigatus*, *Staphylococcus aureus*, *Enterococcus faecalis*, *E. coli*, *Klebsiella* sp., *Candia albicans*		0.6–1 mg/mL	Ethanol, pet ether, chloroform ext. was more active against *E.faecalis* (MIC 0.298, 0.254, and 0.589 mg/mL, respectively)	[18]
	Whole plant	MeOH ext.	In vitro	*E. coli*, *P. aeruginosa*, *Enterobacter aerogenes Providencia stuartii*, *K.pneumoniae*, *Enterobacter cloacae*, *S. aureus*	-	256 µg/mL	inhibition of *S. aureus,* E. coli, *P. aeruginosa, E. aerogenes, K. pneumoniae* (MIC ranges of 64–289 μg/mL	[42]
**Larvicidal Activity**	Seeds	MeOH ext.	In vitro	Larvicidal activity against *Aedes aegypti* and *Culex pipiens pallens*	-	10–300 ppm	40 ppm	[43]
	Seeds	Chloroform fr.	In vitro	Larvicidal activity against *A. aegypti*, *Aedes togoi*, and *Cx. pipiens*	-	25 mg/L	100% Mortality (at concentration 25 mg/L)	[44]
	Leaf	EtOH ext.	In vitro	Larvicidal activity against *Anopheles stephensi*	-	25–125 mg/L	LC_50_ = 52.2 mg/L, LC_90_ = 108.7 mg/L (at concentration 25 mg/L)	[45]
	Leaf	EtOH ext.	In vitro	Anti-oviposition activity against *Anopheles stephensi*	-	100–400 mg/L	92.5% for 400 mg/L 87.2% for 300 mg/L 83.0% for 200 mg/L	[45]

**Table 3:** Major Phytochemicals in *Cassia obtusifolia* and their pharmacological activities.

Compounds	Biological Activity	In Vivo/ In Vitro	Model	Administration (In Vivo)	Dose Range	Active Concentration	Reference
**Anthraquinones**							
**Emodin**	Anti-Alzheimer’s activity	In vitro	(a) Acetylcholinesterase inhibitory activity (b) Butyrylcholinesterase inhibitory activity (c) β-secretase inhibitory activity	-	0–100 µg/mL	(a) IC_50_ = 9.17µg/mL (b) IC_50_ = 157 µg/mL (c) IC_50_ = 4.48 µg/mL	[10]
	Antimicrobial activity	In vitro	Antibacterial activity against (a) *Staphylococcus aureus* 209P (b) *Escherichia coli* NIHJ	-	0–1 mg/mL	MIC (a) 4.5 µg/mL (b) 25 µg/mL	[46]
	Antidiabetic activity	In vitro	(a) PTP 1B inhibitory activity (b) α-glucosidase inhibitory activity (c) Stimulation of glucose uptake in HepG2 cells	-	(a) 0–100 µg/mL (b) 0–400 µg/mL (c) 3.12–12.5 µM	(a) IC_50_ = 3.51 µg/mL (b) IC_50_ = 1.02 µg/mL (c) glucose uptake	[9]
	Platelet anti-aggregatory activity	In vitro	(a) Adenosine 5′-diphosphate inhibitory activity (b) Arachidonic-acid inhibitory activity (c) Collagen inhibitory activity	-	0–1 mg/mL	1 mg/mL	[47]
	Larvicidal activity	In vitro	Larvicidal activity against (a) *Culex pipiens pallens* (b), *Aedes aegypti* (c) *Aedes togoi*	-	1–20 mg/L	(a) LC_50_ = 1.4 mg/L (b) LC_50_ = 1.9 mg/L (c) LC_50_ = 2.2 mg/L	[44]
	Hepatoprotective activity	In vitro	Protection against *t*-BHP-induced hepatotoxicity in HepG2 cells	-	25 µM	protect cells damage	[37]
	Parkinson’s disease activity	In vitro	(a) MAO-A inhibitory activity (b) MAO-B inhibitory activity	-	25 µM	(a) IC_50_ = 23 µM (b) IC_50_ = 54 µM	[19]
**Alaternin**	Neuroprotective activity	In vivo	Prevented nitrotyrosine and lipid peroxidation, as well as BCCAO induced-iNOS expression and significantly reduced microglial activation	Orally	1, 10 mg/kg	10 mg/kg	[48]
	Antidiabetic activity	In vitro	(a) PTP 1B inhibitory activity (b) α-glucosidase inhibitory activity (c) Stimulation of glucose uptake in HepG2 cells	-	(a) 0–100 µg/mL (b) 0–400 µg/mL (c) 12.5–50 µM	(a) IC_50_ = 1.22 µg/mL (b) IC_50_ = 0.99 µg/mL (c) glucose uptake	[9]
	Anti-Alzheimer’s activity	In vitro	(a) Acetylcholinesterase inhibitory activity (b) Butyrylcholinesterase inhibitory activity (c) β-secretase inhibitory activity	-	0–100 µg/mL	(a) IC_50_ = 6.29 µg/mL (b) IC_50_ = 113 µg/mL (c) IC_50_ = 0.94 µg/mL	[10]
	Hepatoprotective activity	In vitro	Protection against *t*-BHP-induced hepatotoxicity in HepG2 cells	-	50, 100 µM	(a) protect cells damage (b) increased GSH level and reduce ROS level	[37]
	Parkinson’s disease activity	In vitro	(a) MAO-A inhibitory activity (b) MAO-B inhibitory activity	-	10 µM	(a) IC_50_ = 5.35 µM (b) IC_50_ = 4.55 µM	[19]
**Obtusifolin**	Neuroprotective activity	In vivo	Significantly reversed scopolamine-induced cognitive impairments in the passive avoidance test, improved escape latencies, swimming times in the target quadrant, and crossing numbers in the zone in Morris water maze test	Orally	0.25–2 mg/kg	0.5 mg/kg	[49]
	Hyperlipidemia and antioxidant activity	In vivo	Reduced body weight, TC, TG, LDL-C and increased HDL-C levels, as well as increased SOD and NO, and reduced MDA levels in hyperlipidemic rats.	Orally	5 and 20 mg/kg	20 mg/kg	[50]
	Neuropathic and anti-inflammatory activity	In vivo	Inhibition of TNF-*α*, IL-1*β*, IL-6 and NF-kB up-regulation in the spinal cord in mice and rat models	Intraperitoneal injection	0.25–2 mg/kg	1 and 2 mg/kg	[51]
	Anti-Alzheimer’s activity	In vitro	(a) Acetylcholinesterase inhibitory activity (b) Butyrylcholinesterase inhibitory activity (c) β-secretase inhibitory activity	-	0–100 µg/mL	(a) IC_50_ = 18.5 µg/mL (b) IC_50_ = 284 µg/mL (c) IC_50_ = 64.8 µg/mL	[10]
	Antidiabetic activity	In vitro	(a) PTP 1B inhibitory activity (b) α-glucosidase inhibitory activity	-	(a) 0–100 µg/mL (b) 0–400 µg/mL	(a) IC_50_ = 35.2 µg/mL (b) IC_50_ = 142 µg/mL	[9]
	Hepatoprotective activity	In vitro	Protection against tacrine-induced hepatotoxicity in HepG2 cells	-	160 µM	Protection ratio value 41.2% at 160 µM	[36]
	Parkinson’s disease activity	In vitro	(a) MAO-A inhibitory activity; (b) MAO-B inhibitory activity	-	100 µM	(a) IC_50_ = 31 µM (b) IC_50_ ≥ 400 µM	[19]
**Gluco-obtusifolin**	Neuropathic and anti-inflammatory activity	In vivo	Inhibition of TNF-*α*, IL-1*β*, IL-6 and NF-kB up-regulation in the spinal cord in mice and rat models	Intraperitoneal injection	0.25–2 mg/kg	1 and 2 mg/kg	[51]
	Anti-Alzheimer’s activity	In vitro	(a) Acetylcholinesterase inhibitory activity (b) Butyrylcholinesterase inhibitory activity (c) β-secretase inhibitory activity	-	0–400 µg/mL	(a) IC_50_ = 37.2 µg/mL (b) IC_50_ = 172 µg/mL (c) IC_50_ = 41.1 µg/mL	[10]
	Neuroprotective activity	In vivo	Significantly reversed scopolamine-induced cognitive impairments in the passive avoidance test, improved escape latencies, swimming times in the target quadrant, and crossing numbers in the zone in the Morris water maze test	Orally	0.25–2 mg/kg	0.5 mg/kg	[49]
	Antidiabetic activity	In vitro	(a) PTP 1B inhibitory activity (b) α-glucosidase inhibitory activity	-	(a) 0–100 µg/mL (b) 0–400 µg/mL	(a) IC_50_ = 53.35 µg/mL (b) IC_50_ = 23.77 µg/mL	[9]
	Platelet anti-aggregatory activity	In vitro	(a) Adenosine 5′-diphosphate inhibitory activity (b) Arachidonic-acid inhibitory activity (c) Collagen inhibitory activity	-	0–1 mg/mL	(a) IC_50_ = 0.25 µg/mL (b) IC_50_ = 0.05 µg/mL (c) IC_50_ = 0.1 µg/mL	[5]
	Parkinson’s disease activity	In vitro	(a) MAO-A inhibitory activity (b) MAO-B inhibitory activity	-	500 µM	(a) IC_50_ ≥ 400 µM (b) IC_50_ ≥ 400 µM	[19]
**Aurantio-obtusin**	Hepatoprotective activity	In vitro	Protection against tacrine-induced hepatotoxicity in HepG2 cells	-	160 µM	Protection ratio value 55.3% at 160 µM	[36]
	Anti-Alzheimer’s activity	In vitro	(a) Acetylcholinesterase inhibitory activity (b) Butyrylcholinesterase inhibitory activity (c) β-secretase inhibitory activity	-	0–100 µg/mL	(a) IC_50_ = 92.1 µg/mL (b) IC_50_ = 314 µg/mL (c) IC_50_ = 67.9 µg/mL	[10]
	Platelet anti-aggregatory activity	In vitro	(a) Adenosine 5′-diphosphate inhibitory activity (b) Arachidonic-acid inhibitory activity (c) Collagen inhibitory activity	-	0–1 mg/mL	1 mg/mL	[48]
	Antidiabetic activity	In vitro	(a) PTP 1B inhibitory activity (b) α-glucosidase inhibitory activity	-	(a) 0–100 µg/mL (b) 0–400 µg/mL	(a) IC_50_ = 27.19 µg/mL (b) IC_50_ = 41.20 µg/mL	[9]
	Anti-cancer activity	In vitro	Cytotoxicity against (a) HCT-116, (b) A549, (c) SGC7901 and (d) LO2 cell lines	-	0.4–50 µg/mL	(a) IC_50_ = 18.9 µg/mL (b) IC_50_ = 20.1 µg/mL (c) IC_50_ = 22.0 µg/mL (d) IC_50_ = 23.1 µg/mL	[52]
	Prevention of bone disease	In vitro	Stimulates osteoblast migration, differentiation, and mineralization in a dose-dependent manner in MC3T3-E1 osteoblast cells	-	0.1–100 µM	10 µM	[53]
	Anti-inflammatory activity	In vitro	(a) Significantly decreased the production of NO, PGE2, and inhibited the iNOS, COX-2, TNF-α and IL-6. (b) Reduced the LPS-induced activation of nuclear factor-κB in RAW264.7 cells.	-	6.12–100 µM	6.12–100 µM	[54]
	Parkinson’s disease activity	In vitro	(a) MAO-A inhibitory activity (b) MAO-B inhibitory activity	-	200 µM	(a) IC_50_ = 27.23 µM (b) IC_50_ = 174.40 µM	[19]
**Obtusin**	Antidiabetic activity	In vitro	(a) PTP 1B inhibitory activity (b) α-glucosidase inhibitory activity	-	(a) 0–100 µg/mL (b) 0–400 µg/mL	(a) IC_50_ = 6.44 µg/mL (b) IC_50_ = 20.92 µg/mL	[9]
	Anti-Alzheimer’s activity	In vitro	(a) Acetylcholinesterase inhibitory activity (b) Butyrylcholinesterase inhibitory activity (c) β-secretase inhibitory activity	-	0–100 µg/mL	(a) IC_50_ = 82 µg/mL (b) IC_50_ = 287 µg/mL (c) IC_50_ = 61.9 µg/mL	[10]
	Anti-cancer activity	In vitro	Cytotoxicity against (a) HCT-116, (b) A549, and (c) SGC7901 cell lines	-	0.4–50 µg/mL	(a) IC_50_ = 13.1 µg/mL (b) IC_50_ = 29.2 µg/mL (c) IC_50_ = 15.2 µg/mL	[52]
	Parkinson’s disease activity	In vitro	(a) MAO-A inhibitory activity (b) MAO-B inhibitory activity	-	400 µM	(a) IC_50_ = 11.12 µM (b) IC_50_ ≥ 400 µM	[19]
**Chryso-obtusin**	Anti-cancer activity	In vitro	Cytotoxicity against (a) HCT-116, (b) A549, (c) SGC7901 and (d) LO2 cell lines	-	0.4–50 µg/mL	(a) IC_50_ = 10.5 µg/mL (b) IC_50_ = 14.6 µg/mL (c) IC_50_ = 12.0 µg/mL (d) IC_50_ = 15.8 µg/mL	[52]
	Anti-Alzheimer’s activity	In vitro	(a) Acetylcholinesterase inhibitory activity (b) Butyrylcholinesterase inhibitory activity (c) β-secretase inhibitory activity	-	0–100 µg/mL	(a) IC_50_ = 68.6 µg/mL (b) IC_50_ = 287 µg/mL (c) IC_50_ = 49.9 µg/mL	[10]
	Antidiabetic activity	In vitro	(a) PTP 1B inhibitory activity (b) α-glucosidase inhibitory activity	-	(a) 0–100 µg/mL (b) 0–400 µg/mL	(a) IC_50_ = 14.88 µg/mL (b) IC_50_ = 36.1 µg/mL	[9]
	Platelet anti-aggregatory activity	In vitro	(a) Adenosine 5′-diphosphate inhibitory activity (b) Arachidonic-acid inhibitory activity (c) Collagen inhibitory activity	-	0–1 mg/mL	1 mg/mL	[47]
	Parkinson’s disease activity	In vitro	(a) MAO-A inhibitory activity (b) MAO-B inhibitory activity	-	400 µM	(a) IC_50_ = 327.67 µM (b) IC_50_ ≥ 400 µM	[19]
**Questin**	Antimicrobial activity	In vitro	Antibacterial activity against (a) *Staphylococcus aureus* 209P and (b) *Escherichia coli* NIHJ	-	0–100 µg/mL	MIC (a) 25 µg/mL (b) 50 µg/mL	[48]
	Anti-Alzheimer’s activity	In vitro	(a) Acetylcholinesterase inhibitory activity (b) Butyrylcholinesterase inhibitory activity (c) β-secretase inhibitory activity	-	0–100 µg/mL	(a) IC_50_ = 34.0 µg/mL (b) IC_50_ = 138 µg/mL (c) IC_50_ = 32.8 µg/mL	[10]
	Antidiabetic activity	In vitro	(a) PTP 1B inhibitory activity (b) α-glucosidase inhibitory activity	-	(a) 0–100 µg/mL (b) 0–400 µg/mL	(a) IC_50_ = 5.69 µg/mL (b) IC_50_ = 136.1 µg/mL	[9]
	Parkinson’s disease activity	In vitro	(a) MAO-A inhibitory activity (b) MAO-B inhibitory activity	-	20 µM	(a) IC_50_ = 0.17 µM (b) IC_50_ = 10.58 µM	[19]
**Gluco-aurantio-obtusin**	Platelet anti-aggregatory activity	In vitro	(a) Adenosine 5′-diphosphate inhibitory activity (b) Arachidonic-acid inhibitory activity (c) Collagen inhibitory activity	-	0–1 mg/mL	(a) IC_50_ = 0.25 µg/mL (b) IC_50_ = 0.05 µg/mL (c) IC_50_ = 0.1 µg/mL	[5]
	Anti-Alzheimer’s activity	In vitro	(a) Acetylcholinesterase inhibitory activity (b) β-secretase inhibitory activity	-	0–100 µg/mL	(a) IC_50_ = 109 µg/mL (b) IC_50_ = 50.9 µg/mL	[10]
	Antidiabetic activity	In vitro	(a) PTP 1B inhibitory activity (b) α-glucosidase inhibitory activity	-	(a) 0–100 µg/mL (b) 0–400 µg/mL	(a) IC_50_ = 31.3 µg/mL (b) IC_50_ = 142.1 µg/mL	[9]
	Hepatoprotective activity	In vitro	Hepatoprotective efficacy against *t*-BHP-induced cell death in HepG2 cells	-	20 µM	Protection ratio value 49.7% at 20 µM	[12]
	Parkinson’s disease activity	In vitro	(a) MAO-A inhibitory activity (b) MAO-B inhibitory activity	-	400 µM	(a) IC_50_ = 39.55 µM (b) IC_50_ = 180.76 µM	[19]
**Chrysophanol; Aloe-emodin; Physcion; Chrysophanol tri, Tetraglucoside; 2-hydroxyemodin-1methylether; Chryso-obtusin-2-*O*-β-d-glucoside**	Antidiabetic activity	In vitro	(a) PTP 1B inhibitory activity (b) α-glucosidase inhibitory activity	-	(a) 0–100 µg/mL (b) 0–400 µg/mL	(a) IC_50_ = 5~103 µg/mL (b) IC_50_ = 5~228 µg/mL	[9]
	Anti-Alzheimer’s activity	In vitro	(a) Acetylcholinesterase inhibitory activity (b) Butyrylcholinesterase inhibitory activity (c) β-secretase inhibitory activity	-	0–400 µg/mL	(a) IC_50_ = 14~71 µg/mL (b) IC_50_ ≥ 100 µg/mL (c) IC_50_ = 13~59 µg/mL	[10]
	Parkinson’s disease activity	In vitro	(a) MAO-A inhibitory activity (b) MAO-B inhibitory activity	-	400 µM	(a) IC_50_ = 2.47~400 µM (b) IC_50_ ≥ 400 µM	[19]
**Dihydroxyanthraquinone**	Bacterial growth promoting and inhibiting activity	In vitro	(a) Growth promoting activity against *Bifidobacterium bifidum* (b) Growth inhibiting activity against *Clostridium perfringens* and *Escherichia coli*	-	(a) 0.05–0.5 mg/d (b) 0.1–5 mg/d	(a) GIR > 2.0 at 0.5 mg/disk (b) Inhibitory zone diameter > 30 mm	[7]
**Naphthopyrone**							
**Cassiaside**	Anti-Alzheimer’s activity	In vitro	(a) Acetylcholinesterase inhibitory activity (b) Butyrylcholinesterase inhibitory activity (c) β-secretase inhibitory activity	-	0–100 µg/mL	(a) IC_50_ = 18.1 µg/mL (b) IC_50_ = 177 µg/mL (c) IC_50_ = 1.85 µg/mL	[10]
	Antidiabetic activity	In vitro	(a) PTP 1B inhibitory activity (b) α-glucosidase inhibitory activity	-	(a) 0–100 µg/mL (b) 0–400 µg/mL	(a) IC_50_ = 48.55 µg/mL (b) IC_50_ = 129.2 µg/mL	[9]
	Hepatoprotective activity	In vitro	Hepatoprotective efficacy against *t*-BHP-induced cell death in HepG2 cells		25 µM	(a) protect cells damage (b) increased GSH level and reduce ROS level	[37]
	Parkinson’s disease activity	In vitro	(a) MAO-A inhibitory activity (b) MAO-B inhibitory activity	-	400 µM	(a) IC_50_ = 11.26 µM (b) IC_50_ ≥ 400 µM	[19]
**Isotoralactone; Toralactone**	Antimicrobial activity	In vitro	Antibacterial activity against (a) *Staphylococcus aureus* 209P and (b) *Escherichia coli* NIHJ	-	0–100 µg/mL	MIC (a) 2–3 µg/Ml (b) 5.5–12 µg/mL	[46]
**Cassiaside B2, Cassiaside C2**	Antiallergic activity	In vitro	Inhibition of histamine release in rat peritoneal mast cells	-	100 µM	Cassiaside B2 inhibit 17.2%; Cassiaside C2 Inhibit 53.9%	[6]
**Toralactone Gentiobioside**	Antidiabetic activity	In vitro	(a) PTP 1B inhibitory activity (b) α-glucosidase inhibitory activity	-	(a) 0–100 µg/mL (b) 0–400 µg/mL	(a) IC_50_ = 81.1µg/mL (b) IC_50_ = 37.60 µg/mL	[9]
	Anti-Alzheimer’s activity	In vitro	(a) Acetylcholinesterase inhibitory activity (b) Butyrylcholinesterase inhibitory activity (c) β-secretase inhibitory activity	-	0–100 µg/mL	(a) IC_50_ = 91.3 µg/mL (b) IC_50_ = 117 µg/mL (c) IC_50_ = 69.0 µg/mL	[10]
	Hepatoprotective activity	In vitro	Hepatoprotective efficacy against *t*-BHP-induced cell death in HepG2 cells	-	20 µM	Increased in Nrf2/ARE-luciferase activity, and upregulated NQO1, GLC, HO-1 levels	[12]
**rubrofusarin, Rubrofusarin 6-*O*-β-d-glucopyranoside, Rubrofusarin 6-*O*-β-d-gentiobioside, Nor-rubrofusarin 6-*O*-β-d-glucoside**	Anti-Alzheimer’s activity	In vitro	(a) Acetylcholinesterase inhibitory activity (b) β-secretase inhibitory activity	-	(a) 0–100 µM (b) 0–750 µM	(a)15.95–148 µM (b) 14.0–190 µM	[55]
